# Integrated transcriptome and metabolome profiling reveals mechanisms underlying the infection of *Cytospora mali* in “Jin Hong” branches

**DOI:** 10.3389/fmicb.2024.1394447

**Published:** 2024-04-24

**Authors:** Jing Zhao, Yuan Guo, Zhengnan Li, Yajun Shi, Pingping Sun

**Affiliations:** ^1^College of Horticulture and Plant Protection, Inner Mongolia Agricultural University, Hohhot, China; ^2^Beijing Engineering Research Center for Edible Mushroom, Institute of Plant Protection, Beijing Academy of Agriculture and Forestry Sciences, Beijing, China

**Keywords:** apple, *Cytospora mali*, transcriptome, metabolome, conjoint analysis

## Abstract

**Introduction:**

Valsa canker, caused by *Cytospora mali*, is a destructive disease in apple production. However, the mechanism by which apple defend against *C. mali* infection remains unclear.

**Methods:**

In this study, the integrative transcriptional and metabolic analysis were used to investigate the responses of the ‘Jin Hong’ apple branches to the invasion of *C. mali*.

**Results and Discussion:**

Results showed that the differentially expressed genes were mainly enriched in the pathways of carbon metabolism, photosynthesis-antenna proteins, and biosynthesis of amino acids pathways. Additionally, the differentially accumulated metabolites were significantly enriched in aminoacyl-tRNA biosynthesis, fructose and mannose metabolism, and alanine, aspartate, and glutamate metabolism pathways. Conjoint analysis revealed that *C. mali* infection significantly altered 5 metabolic pathways, 8 highly relevant metabolites and 15 genes of apples. Among which the transcription factors WRKY and basic domain leucine zipper transcription family were induced, the α-linolenic acid and betaine were significantly accumulated in *C. mali* infected apple stems. This work presents an overview of the changes in gene expression and metabolic profiles in apple under the inoculation of *C. mali*, which may help to further screen out the mechanism of plant-pathogen interaction at the molecular level.

## 1 Introduction

Apple Valsa Canker (AVC), also known as *Cytospora* canker, is caused by the necrotrophic fungus *Cytospora mali* Grove (Yin et al., 2015; Wang D. L. et al., 2020). AVC stands out as one of the most destructive diseases affecting apples (*Malus domestica*) in the world, leading to significant yield losses and decreased fruit quality. Extensive surveys have revealed that the incidence of AVC averaged approximately 53% in the major apple production regions of China, and reached up to 80% in severely affected orchards (Meng et al., 2019).

AVC mainly occurs on the branches of trees, the infected tissues then gradually dry out, collapse slightly, and finally form localized cankers and even kills entire apple trees (Feng et al., 2023). *C. mali* can extensively penetrate into the host’s phloem and xylem, which is difficult for traditional chemical agents to access, and making it harder to prevent (Yin et al., 2015).

The molecular mechanism of apples in responses to *C. mali* infection has been explored in recent years. It is reported that apples could resistant against AVC by negatively regulating the production of phloridzin (Zhou et al., 2019, 2021). Mao et al. (2021) revealed that MdCN11 and MdCN19 genes could negatively regulate the AVC resistance via inducting hypersensitive response. DEGs related to hormonal, Ca^2+^ signaling, and phenylpropanoid biosynthesis were highly expressed to enhance the apple’s resistance to *C. mali* (Yin et al., 2016; Zuo et al., 2018). Wen et al. (2023) established an efficient system for screening disease-resistant genes in response to AVC. In addition, the apple activates and accumulates defense-related enzymes and metabolites to protect itself from *C. mali*, such as phenylalanine ammonia-lyase (PAL), β-1,3-glucanase, and chitinase, dopamine, trans-cinnamic acid, coumarin as well as chlorogenic acid and other phenylpropanoids and flavonoids (Sung and Lee, 2010; Yu et al., 2016; Moneo-Sánchez et al., 2018; Geng et al., 2020; Liu X. M. et al., 2021; Liu X. Z. et al., 2021; Zhao et al., 2021; Du et al., 2023; Li C. R. et al., 2023). In recent years, the metabolomics and transcriptomics have become a research hotspot to explore the molecular mechanisms of disease resistance in apples against pathogens. Zhao et al. (2022) revealed mechanisms of disease resistance in apples induced by *Wickerhamomyces anomalus*, using the integrated transcriptomic and metabonomic analysis. The transcriptomics was used to explore the molecular mechanisms of apples in responses to *C. mali* infection (Feng et al., 2023). However, there are no studies on combined transcriptomics and metabolomics analyses of apples by challenging with *C. mali*, and some highly relevant genes and metabolites remain unexplored.

This work conducted an integrative analysis of the transcriptome and metabolome of apple branches challenging with *C. mali*. Significant changes were observed in both the transcriptional and metabolic profiles. A conjoint analysis was then performed to identify co-enrichment pathways in both transcriptional and metabolic data. A number of altered pathways, as well as highly correlated genes and metabolites, were identified. These findings will deepen our understanding of AVC caused by *C. mali*, and provide valuable insights for further investigation into the molecular mechanisms of apples against AVC.

## 2 Materials and methods

### 2.1 Plant materials and fungal strains

**Figure 1:**
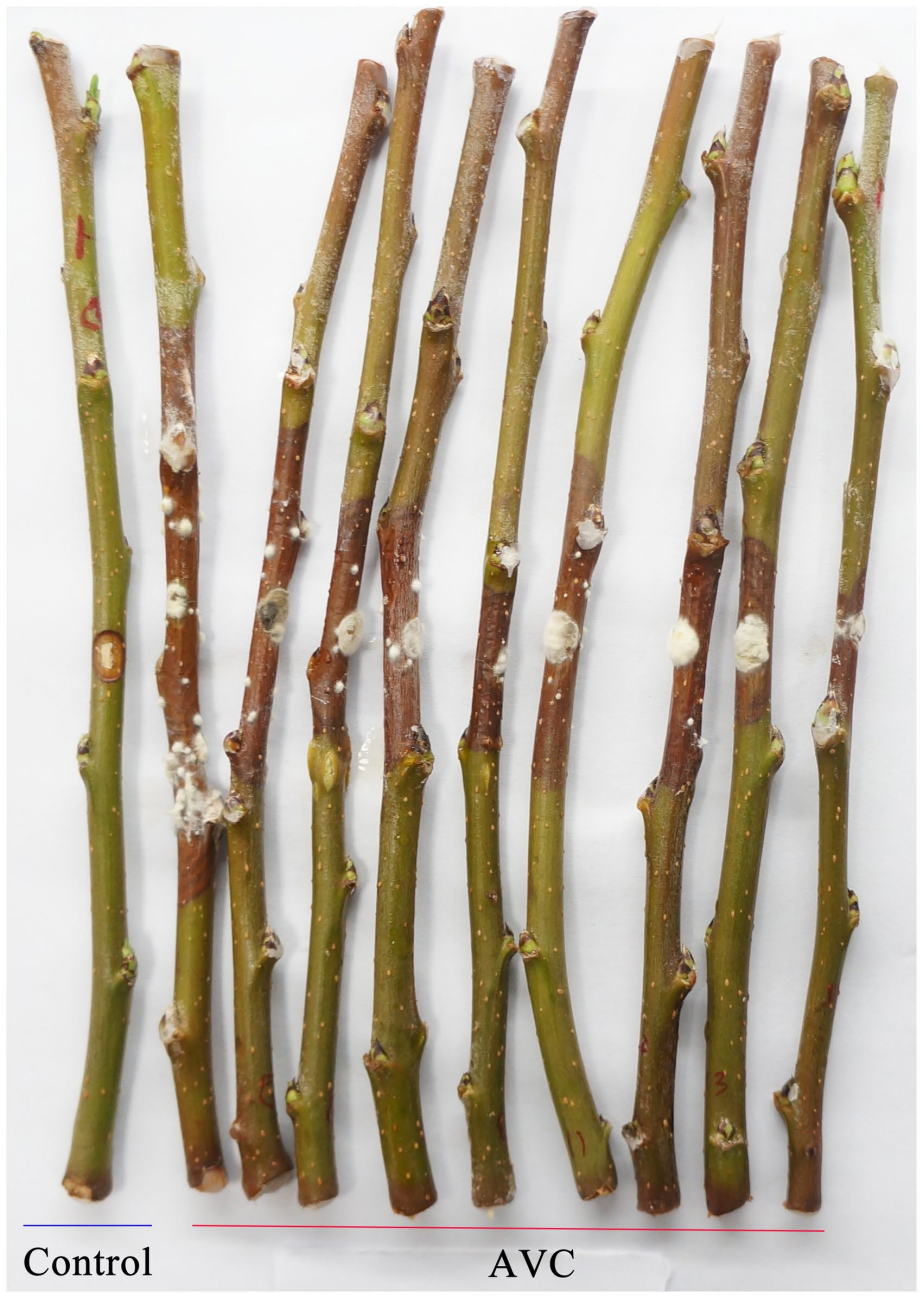
The symptoms of ‘Jin Hong’ apple branches infected with *C. mali*.

*C. mali* (strain QH2), one of the main species causing AVC in northern China (Ma et al., 2020), was used in this study. The strain was isolated from the infected stems of apple tree in Hohhot, Inner Mongolia. The apple branches used in this study were obtained from the orchard in the Inner Mongolia Agricultural University’s campus, the collected branches were inoculated with *C. mali* QH2 following Zang’s method (Zang et al., 2007). Briefly, branches from “Jin Hong” apple tree were cut into 30 cm length, immersed in 1% sodium hypochlorite for 10 min, 75% alcohol for 3 min, and washed with sterile water 3 times. The two ends of the twigs were sealed by dipping in heated liquid wax and the middle of the twigs was scalded by a soldering iron 6 mm in diameter. *C. mali* QH2 disks (Φ = 6 mm) were placed on the scalded area. The fungal disks were wrapped with sterile wet degreasing cotton and preservative film. Healthy branches inoculated with agar were treated as control. Treated branches were placed in a pallet with two players of gauze, and incubated in an illumination incubator with a 16 h light/8 h dark cycle, 85% humidity at 25°C for 9 days (Figure 1). Approximately 2.0 g of bark tissue, including buds, from the borders of infected area was sampled. Each sample was divided into two parts, for transcriptomic and metabolomic analysis, respectively.

### 2.2 Total RNA extraction, library construction, and sequencing

The total RNA was extracted using the Takara Mini BEST plant RNA Extraction Kit (Takara, Kyoto, Japan) followed by the instructions. The RNA quality was assessed using the NanoPhotometer^®^ spectrophotometer (IMPLEN, LA, CA, United States) and the RNA Nano 6000 Assay Kit of the Bioanalyzer 2100 system (Agilent Technologies, Palo alto, CA, United States).

A total of 1 μg RNA per sample was used for RNA sample preparation. The NEBNext^®^ UltraTM RNA Library Prep Kit for Illumina^®^ (NEB, Ipswich, MA, USA) was used to generate sequencing libraries. Index codes were added to attribute sequences to each sample.

Briefly, mRNA was enriched by poly A tail selection and chemically fragmented. Subsequently, second strand cDNA synthesis was performed using DNA Polymerase I and RNase H. The synthesized cDNA was then subjected to end-repair and “A” base addition according to Illumina’s library construction protocol. To select cDNA fragments for PCR, we purified the library fragments using AMPure XP beads (Beckman Coulter, Beverly, United States) to ensure a length of 250–300 bp. We also purified the PCR products and assessed the library quality on the Agilent Bioanalyzer 2100 system. Following cluster generation, we sequenced the library preparations on an Illumina Novaseq platform, generating 150 bp paired-end reads.

### 2.3 Data quality control

We performed splicing and quality control on the raw data. Aligned paired-end clean reads to the reference genome using HISAT2 v2.0.5. Used Feature Counts (1.5.0-p3) to calculate the number of reads mapped to each gene. Then calculated the fragments per kilobase of transcript per million mapped reads (FPKM) for each gene based on gene length and determine the number of reads mapped to that gene.

### 2.4 Differential expression analysis

Differentially expressed genes (DEGs) were identified using the DESeq2 R package (version 1.16.1) (Varet et al., 2017). Differential expression analysis of two conditions was performed using the edgeR R package (version 3.18.1) (Dietz et al., 2010; McCarthy et al., 2012; Varet et al., 2017).

The resulting p were adjusted using the Benjamini and Hochberg’s approach (Yoav and Yosef, 2000) to control for false discovery rate. DEGs were determined according to the following general rules: |Log2FoldChange(FC)| > 1 and *p*-adjust ≤0.05. Gene ontology (GO) and Kyoto Encyclopedia of Genes and Genomes (KEGG) pathway functional enrichment analyses were performed using the clusterProfiler R package (version 3.4.4) (Wu et al., 2021).

### 2.5 Metabolite extraction

The metabolite was extracted using a solution of methanol/acetonitrile/water in a 2: 2: 1 (v/v) ratio. The mixture was vortexed and subjected to low-temperature sonication for 30 min and stand at −20°C for 10 min, then centrifuged (Eppendorf, 5430R, Hamburg, Germany) at 14,000 g at 4°C for 20 min, the supernatant was collected and vacuum dried. For mass spectrometry analysis, 100 μL of acetonitrile/water solution (acetonitrile: water = 1: 1, v/v) was added for reconstitution, followed by vertexing, the reconstituted sample was then centrifuged at 14,000 g for 15 min at 4°C to obtain the supernatant.

### 2.6 Non-target metabolomics

Metabolites were chromatographically separated using an ultra-high performance liquid chromatography (UHPLC) system (Agilent 1290 Infinity LC, Agilent Technologies, Palo Alto, California, United States). The UHPLC system was equipped with an ACQUITY BEH C18 column (100 mm × 2.1 mm column, 1.7 μm, Waters, Milford, MA, United States). The mobile phases consisted of solvent A (Water +25 mM ammonium acetate +25 mM ammonia) and solvent B (acetonitrile). The gradient elution program was set as follows to equilibrate the systems: from 0 to 0.5 min, 95% (B); from 0.5 to 7 min, 95 to 65% (B); from 7 to 8 min, 65 to 40% (B); from 8 to 9 min, 40% (B); 9 to 9.1 min, 40 to 95% (B); from 9.1 to 12 min, 95% (B). The sample injection volume was 2 μL, the flow rate was 0.5 mL min^−1^, and the column temperature was maintained at 25°C throughout the chromatographic separation.

Mass spectra of the compounds were obtained using an AB Triple TOF 6600 (AB SCIEX, Framingham, United States). ESI source conditions after HILIC chromatographic separation Ion source Gas1 (Gas1): 60, Ion source Gas2 (Gas2): 60, curtain gas (CUR): 30, source temperature: 600°C Ion source voltage floating (ISVF) ± 5,500 V; TOF MS scan m/z range: 60–1,000 Da, product ion scan m/z range: 25–1,000 Da, TOF MS scan accumulation time 0.20 s/spectra, product ion scan accumulation time 0.05 s/spectra; MS2 data were acquired using information dependent acquisition (IDA) and operated in high sensitivity mode, declustering potential (DP): ± 60 V, collision energy: 35 ± 15 eV, exclude isotopes within 4 Da, candidate ions to monitor per cycle: 10. Samples were analyzed in both positive (+) and negative (−) ESI modes.

Raw data in Wiff format were converted to mzXML format using ProteoWizard (Darren et al., 2008) and then peaks were aligned and peak areas extracted using XCMS software (Domingo-Almenara and Siuzdak, 2020). The data extracted by XCMS were used for metabolite structure identification and data pre-processing, quality evaluation, and multi-variate analysis.

### 2.7 Differentially accumulated metabolites analysis

Orthogonal partial least squares discriminant analysis (OPLS–DA) was used to determine the differentially accumulated metabolites between pairwise groups. The *p* was estimated using a Hotelling’s T2 test for statistical analysis. Differentially accumulated metabolites (DAMs) (VIP ≥ 1, |Log2FC| > 1) between groups were mapped into biochemical pathways using metabolic enrichment and pathway analysis based on MetaboAnalyst.^1^ Those pathways with *p* ≤ 0.05 were considered significantly enriched.

### 2.8 Statistical analysis

OPLS–DA was performed using SIMCA 14.1 (Umetrics, Umea, Sweden). The data were log-transformed. The reliability of the predictive models was assessed using the analysis of variance test of cross-validated predictive residuals (CV–ANOVA), the coefficient of determination (R2) and the predicted variance (Q2) which is the proportion of the total variation of X or Y that can be predicted by a component. To test the overall associations between DEGs and DAMs, the DEGs and DAMs co-enriched in KEGG pathways were identified by KEGG analysis. The DEGs and DAMs were subjected to the integration analysis using regularized canonical correlation analysis (rCCA) (Cruz-Cano and Lee, 2014) to explore their relationships. rCCA was implemented with mixOmics (Rohart et al., 2017) using the R program. The regularization parameters (lambda1, lambda2) were optimized using the *tune.rcc* function. The network communities were determined using the Fast Greedy algorithms with the clusterMaker program (Morris et al., 2011; Utriainen and Morris, 2023) and visualized using Cytoscape 3.10.1 (Zhou et al., 2022). Network attributes were calculated using Cytoscape’s built-in network analyzer. Bioinformatics analysis was performed using dynamic real-time interactive online platforms including APPLIED PROTEIN TECHNOLOGY,^2^ Metware Cloud,^3^ Majorbio,^4^ Omicsmart,^5^ and bioinformatics.^6^

## 3 Results

### 3.1 Transcriptional profiling reveals altered pathways and genes in diseased apple branches

**Figure 2:**
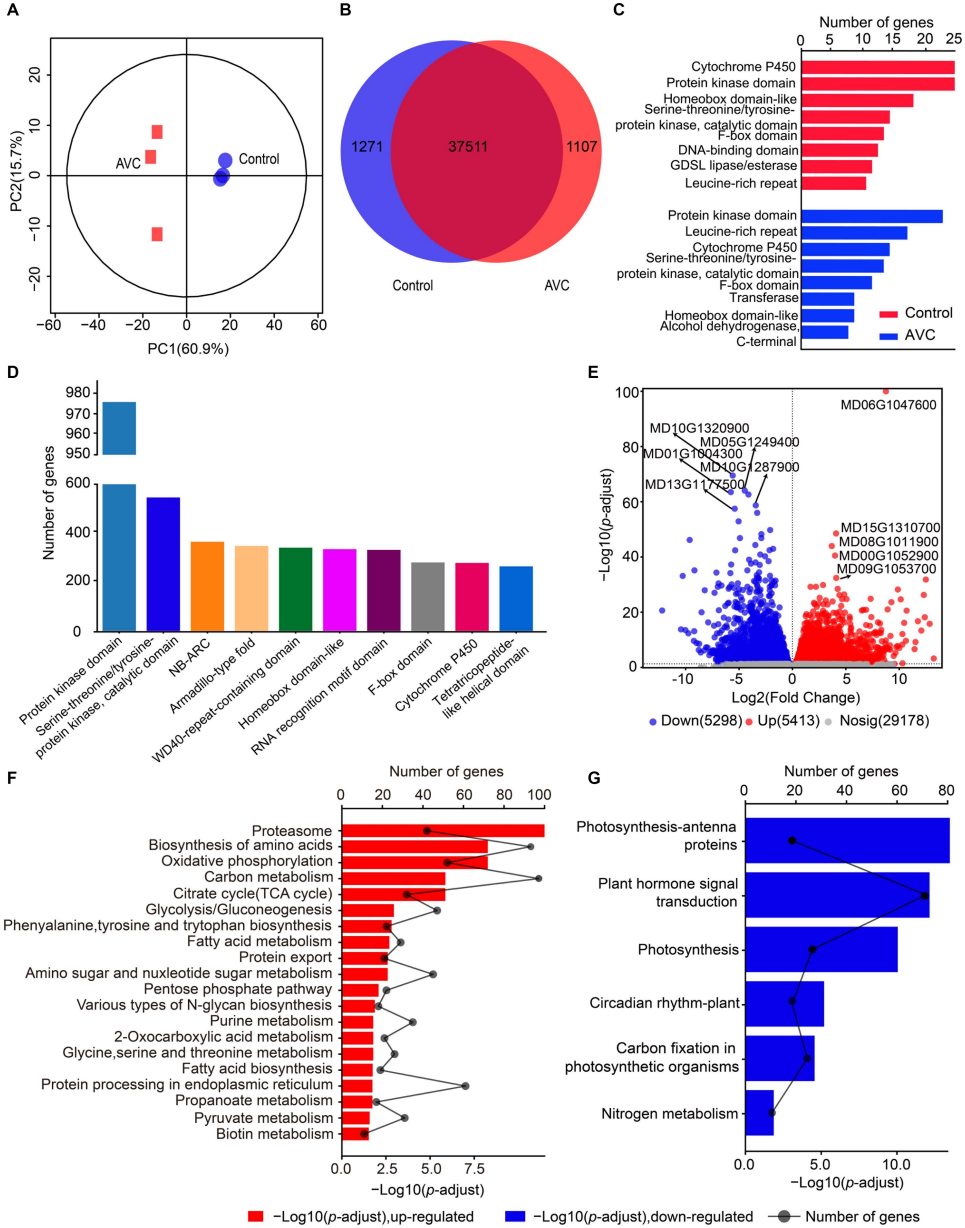
Gene expression analysis of “Jin Hong” in response to AVC infection. **(A)** Principal component analysis (PCA) of transcriptome data. **(B)** The venn plot of differentially expressed genes. **(C)** The unique genes of two groups. **(D)** The genes shared by both groups. **(E)** The volcano plot showing the up-and down-regulated genes (*p*-adjust < 0.05, |Log2FC| > 1, red represents up-regulated genes, blue represents down-regulated genes, and gray indicates not significantly expressed genes, text highlighted for up-and down-regulated Log2FC top 5 genes). **(F,G)** The KEGG enriched analysis of the up-and down-regulated genes in AVC _vs_ Control (*p*-adjust < 0.05).

The transcriptome datasets generated in this work are deposited in the NCBI repository with the accession number: PRJNA1074127, following the link: https://www.ncbi.nlm.nih.gov/sra/PRJNA1074127. The PCA analysis revealed that the two principal components explained 60.9 and 15.7% of the variation and effectively separated samples from different stages (Figure 2A). The Venn analysis showed that 1,271 genes were unique to the control group, 1,107 genes were unique to AVC, and 37,511 genes were shared between the two groups (Figure 2B). The genes related to cytochrome P450, protein kinase domain, and homeobox domain-like were the most prevalent among control-specific genes. Among AVCs, the highest percentage of genes were for protein kinase domain, leucine-rich repeat, and cytochrome P450 (Figure 2C). The most predominant genes shared by both groups were for protein kinase domain, serine–threonine/tyrosine-protein kinase, and catalytic domain (Figure 2D). The results indicated that the expression of 5, 413 DEGs was up-regulated and that of 5, 298 DEGs was down-regulated in the AVC group compared to the control group (Figure 2E). The DEGs identified were annotated using KEGG enrichment analysis. The up-and down-regulated DEGs were enriched in different pathways. The up-regulated genes were mostly enriched in the proteasome, biosynthesis of amino acids and oxidative phosphorylation. On the other hand, the down-regulated genes were enriched in photosynthesis—antenna proteins, plant hormone signal transduction and photosynthesis (Figures 2F,G). The KEGG analysis of all DEGs revealed that the pathways were significantly involved in carbon metabolism, photosynthesis-antenna proteins, and biosynthesis of amino acids (Supplementary Table S1).

After performing GO analysis on the up-regulated genes, the most significantly enriched molecular function (MF) were threonine-type endopeptidase activity and threonine-type peptidase activity. Additionally, the most significantly enriched cellular component (CC) was organelle membrane. Moreover, the down-regulated mRNAs indicate significant enrichment in thylakoid, thylakoid part, and photosynthetic membrane (Supplementary Table S2).

### 3.2 Metabolic profiling reveals altered pathways and metabolites in diseased apple branches

**Figure 3:**
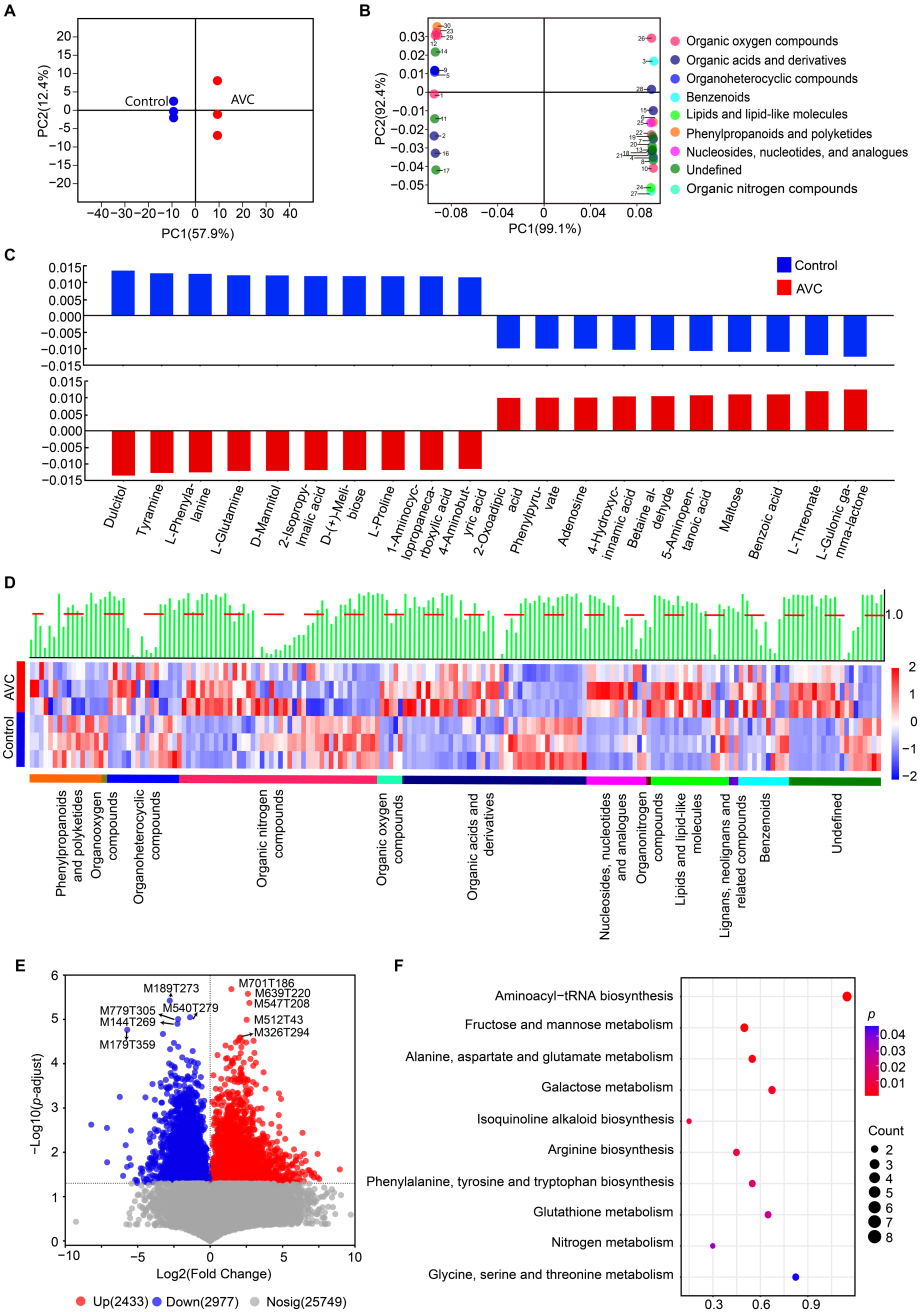
Metabolic profiling of “Jin Hong” in response to AVC infection. **(A)** OPLS–DA analysis of the metabolome data of all samples (R2Xcum = 0.764, R2Ycum = 0.980, Q2cum = 0.969, CV–ANOVA, *p* = 5.577 × 10^−3^). **(B)** OPLS–DA loading showing the top 30 metabolites (VIP > 1), color indicates the superclass. **(C)** Correlation coefficient plots of OPLS–DA showing the relationship between the X and Y variables for the predictive components. The size of the scaled coefficient represents the change in the Y variable when the X variable varies from 0 to 1 in coded units (Selection of the top 5 substances with positive and negative correlation coefficients). **(D)** The heatmap shows the qualitative names of metabolites, the green bars represent the VIP values of the metabolites and the red dashed line indicates VIP = 1, different colored lines indicate the superclass classification of metabolites. **(E)** The volcano plot of DAMs in ‘Jin Hong’ upon exposure to AVC (*p* < 0.05, |Log2FC| > 1, red represents up-regulated metabolites, blue represents down-regulated metabolites, and gray indicates not significantly expressed genes, text highlighted for up-and down-regulated|Log2FC|top 5 metabolites). **(F)** The top 10 enriched KEGG pathways of DAMs in “Jin Hong” upon exposure to AVC infection (*p* < 0.05).

A summary table highlighting the expressed metabolites can be found in Supplementary Table S3. The OPLS-DA analysis showed a significant difference between the control and AVC groups (Figure 3A). The identified metabolites were annotated using the HMDB database and classified into the following superclass categories: 16 phenylpropanoids and polyketides, 1 organooxygen compound, 16 organoheterocyclic compounds, 43 organic oxygen compounds, 5 organic nitrogen compounds, 40 organic acids and derivatives, 13 nucleosides, nucleotides, and analogs, 1 organonitrogen compound, 17 lipids and lipid-like molecules, 2 lignans, neolignans and related compounds, 11 benzenoids, and 20 metabolites of unknown classification. The top 30 metabolites, as shown in the loading plot (VIP > 1), were classified into 9 superclass categories (Figure 3B). The bar charts illustrated the significant regression coefficients of each compound for the different groups (Figure 3C). The number of compounds with significant correlations differed between the two groups. The metabolites of benzoic acid, L-threonate and L-gulonic gamma-lactone were positively correlated with the AVC group, while the metabolites of dulcitol, tyramine, and l-phenylalanine showed a negative correlation. Heatmap showed different superclasses of identified metabolites (Figure 3D). The volcano plot illustrated the DAMs between AVC and control. There were 2,433 up-regulated and 2,977 down-regulated DAMs (Figure 3E). The DAMs between the two groups were mapped to the KEGG database. The identified 48 DAMs were mainly enriched in pathways of aminoacyl-tRNA biosynthesis, fructose and mannose metabolism, alanine, aspartate and glutamate metabolism, galactose metabolism, and isoquinoline alkaloid biosynthesis (Figure 3F; Supplementary Table S4).

### 3.3 Comparative transcriptional and metabolic profiling revealed co-enrichment of pathways

**Figure 4:**
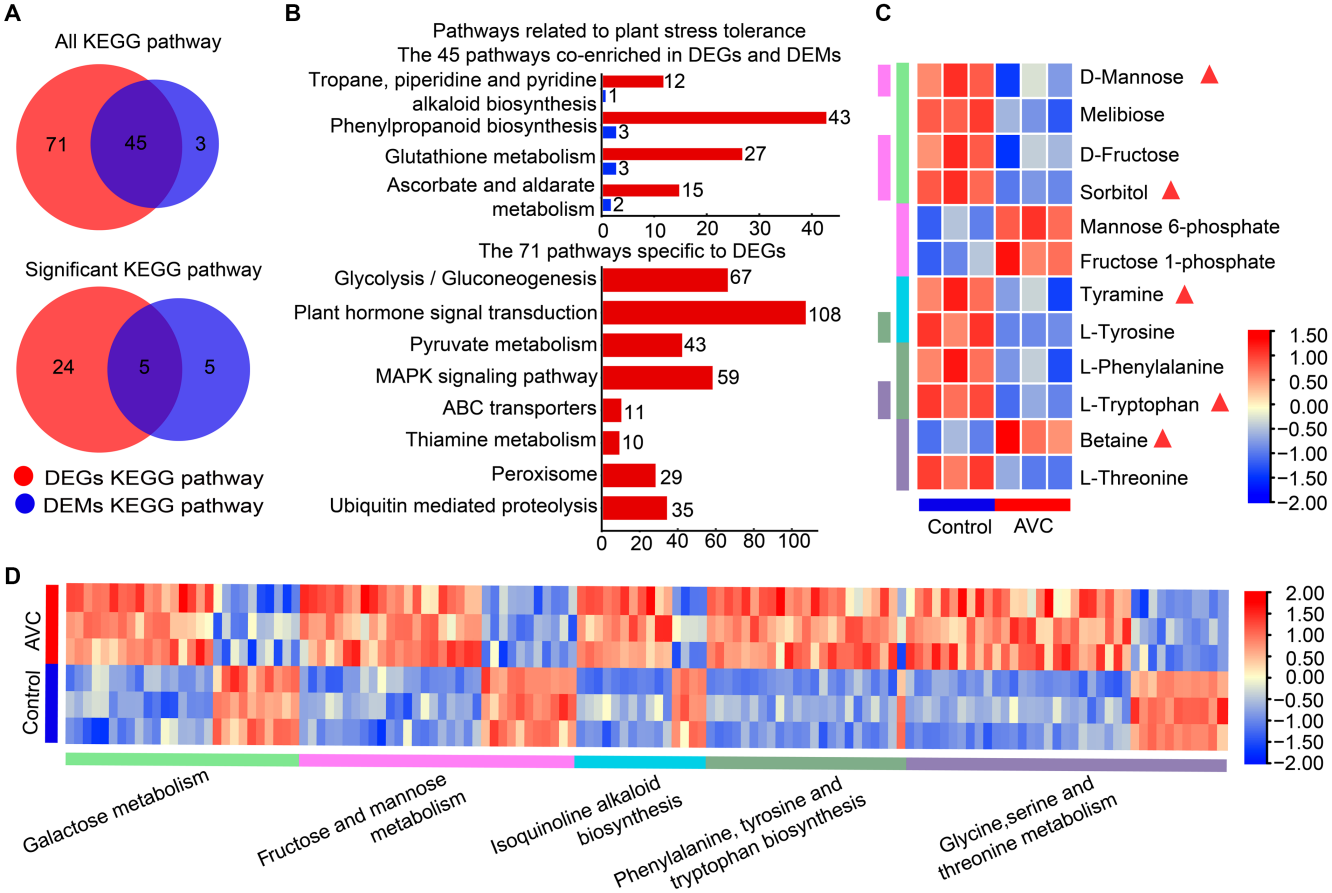
Transcriptome-metabolome-wide association of KEGG pathways. **(A)** The venn plot of DEGs and DAMs enriched all KEGG pathways and significant KEGG pathways. **(B)** The pathways associated with plant stress resistance among the 45 commonly enriched pathways as well as those unique to DEGs. **(C,D)** The heatmap of metabolites and genes among the 5 commonly significant enriched pathways, the triangles are labeled with substances associated with plant stress tolerance and the different colored columns indicate KEEG pathways.

Venn analysis results indicated that DEGs and DAMs were both enriched in 45 KEGG metabolic pathways, among which, 5 were significantly enriched (*p* < 0.05) (Figure 4A). Figure 4B illustrated pathways associated with plant stress tolerance, including 4 of the 45 co-enriched KEGG pathways and 8 pathways specific to DEGs. The DEGs and DAMs were significantly enriched in 5 KEGG pathways, including galactose metabolism, fructose and mannose metabolism, phenylalanine, tyrosine and tryptophan biosynthesis, glycine, serine and threonine metabolism, and isoquinoline alkaloid biosynthesis. Some metabolites are associated with plant resistance to adversity stress, and the metabolites in the 5 significantly enriched shared pathways contain sorbitol, D-mannose, tyramine, L-tryptophan, and betaine (Figures 4C,D).

### 3.4 Combined transcriptional and metabolic profiling revealed relevant genes and metabolites

**Figure 5:**
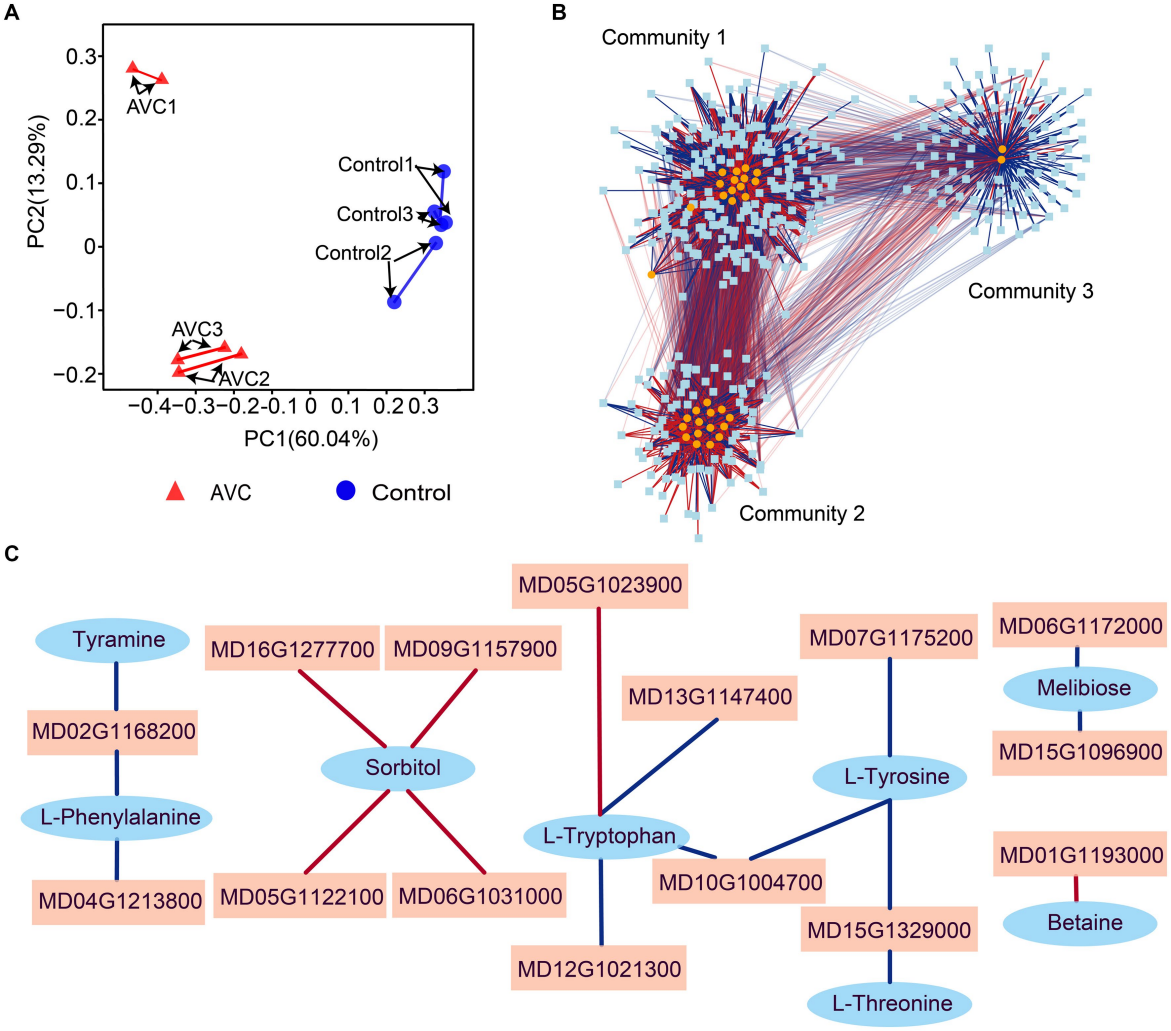
Transcriptome-metabolome-wide association network of all samples. **(A)** Procrustes analysis between all genes and metabolites from all samples. **(B)** Integration network of highly association genes and metabolites at threshold of 0.9. **(C)** Correlation analysis of genes and metabolites in 5 commonly significant pathways (threshold>0.99), blue ovals indicate metabolites, pink squares indicate genes, and red line segments indicate positive correlations and blue line segments indicate negative correlations.

Strong associations were found between genes and metabolites by procrustes analysis (Figure 5A). To identify genes and metabolites that are highly correlated with changes in apples in response to infestation with *C. mali*, the 45 DEGs and DAMs co-enriched pathways were subjected to a correlation analysis using the rCCA method (threshold > 0.90). The correlation network revealed 3 clear gene-metabolite regulatory communities (Figure 5B). In community 1, 94 transcripts exhibited a strong correlation coefficient with L-glutamine and 1-aminocyclopropanecarboxylic acid. Based on the magnitude of the betweenness centrality value, these metabolites were found to be highly correlated with genes MD01G1037400, MD05G1023900, MD14G1102200, MD15G1077000, and MD15G1204200. The interaction networks between the 15 metabolites and 79 transcripts were organized in control and AVC in community 2. The metabolites D-glucuronolactone and alpha-linolenic acid (ALA) had higher connectivity with genes MD00G1112500, MD02G1123600, MD04G1246300, MD10G1063600, and MD00G1061700. Community 3 was the largest gene-metabolite association, consisting of 13 metabolites and 204 genes. The genes MD16G1179500, MD17G1068900, MD07G1159300, MD12G1101800, and MD09G1078700 were found to be more correlated with the metabolites 2-oxoadipate and biotin (Supplementary Table S5). Additionally, we constructed a correlation network of DEGs and DAMs in 5 co-enriched pathways on the Pearson correlation coefficient (PCC) analysis results. Figure 5C showed that 15 DEGs and 8 DAMs in the network were highly correlated (PCC > 0.99). These genes and metabolites may play a crucial role in the defense of apples against AVC.

## 4 Discussion

Almost all apple trees can be infected by AVC (Ke et al., 2014; Haxim et al., 2022). Plants have developed various mechanisms to cope with pathogenic stresses, such as forming physical barriers and activating their immune system, which mainly includes reinforcing the cell wall, producing reactive oxygen species, altering hormone levels and gene expression, activating pathogenesis-related proteins, accumulating defensive metabolites, and inducing the allergic necrosis of plant cells (Yin et al., 2015; Wang X. L. et al., 2020; Liu X. M. et al., 2021; Liu X. Z. et al., 2021; Wang et al., 2022; Xin et al., 2023). In this study, the RNA-seq and UHPLC–MS/MS techniques were utilized to investigate the changes in genes and metabolites in apple branches during *C. mali* infection, 5 metabolic pathways were significantly enriched: galactose metabolism, fructose and mannose metabolism, phenylalanine, tyrosine and tryptophan biosynthesis, glycine, serine and threonine metabolism, and isoquinoline alkaloid biosynthesis. Furthermore, the 15 key genes and 8 DAMs (tyramine, L-tyrosine, L-phenylalanine, sorbitol, L-tryptophan, L-threonine, melibiose, and betaine) that may be associated with plant’s reaction to pathogen stress were identified.

The cell wall provides initial protection and signal perception against pathogen attacks through sensing and regulating related components on the cell wall (Sebastian, 2022; Xin et al., 2023). Fungal pathogens have developed various combinations of plant cell wall degrading enzymes to break them down (Yin et al., 2015; Xin et al., 2023). β-galactosidase (β-Gal) is an essential cell wall glycosyl hydrolase in apple (Yin et al., 2015; Li X. L. et al., 2023). The gene MD02G1287500 encoding β-Gal was found to be down-regulated in the galactose metabolite pathway in this work, this result is consistent with previous results, that apples may slow down the degradation of the cell wall by decreasing the expression of β-Gal to resist pathogen invasion (Moneo-Sánchez et al., 2018; Zhao et al., 2021; Feng et al., 2023).

UDP-glucose catalyzes the glycosylation of phloretin to phloridzin, the pathogen could utilize phloridzin to produce toxins that facilitate necrosis in apple bark (Zhou et al., 2021). Our findings showed a 4-fold down-regulation of the UDP-glucose gene MD13G1093700 in the galactose metabolism pathway and decreased accumulation of its metabolite phenothiazine, although this change was not statistically significant. So this result is consistent with the previous finding that the apples could enhance AVC resistance by decreasing the phloridzin biosynthesis, and the down expression of UDP-glucose genes, which may indirectly modulating cell wall deposition and increasing hormone levels (Zhou et al., 2019, 2021; Feng et al., 2023).

Once the pathogen has penetrated the external cell wall, the intracellular immune system is triggered. Several reports have demonstrated that the WRKY and basic domain leucine zipper (bZIP) gene families play significant roles in various biological processes, including physiological metabolism and stress response (Alves et al., 2013; Jiang et al., 2017), and are particularly involved in resisting *C. mali* in apples (Tsuda and Somssich, 2015; Geng et al., 2020; Liu X. M. et al., 2021; Liu X. Z. et al., 2021; Mao et al., 2021; Wen et al., 2023). Moreover, WRKYs played a crucial role in mediating the communication between jasmonic acid and salicylic acid and are integral to the plant’s defense responses (Phukan et al., 2016; Lui et al., 2017; Han et al., 2023). Transcriptomic analysis in this study revealed that the invasion of *C. mali* activated the expression of WRKY and bZIP genes in apple. There were significant increase in the expression of the WRKY genes (MD13G1122100, MD09G1008800, MD17G113810, and MD03G119760) and bZIP gene (MD08G1025800) in the diseased apple branches. These high expression in WRKY and bZIP genes might associated with the apple’s defense response.

The destruction of cellular membranes in pathogen-affected plants releases a large number of polyunsaturated fatty acids, which act as signal molecules to activate plant defense responses (De Carvalho and Caramujo, 2018). For example, α-linolenic acid (ALA) serves as a precursor to jasmonic acid, a key phytohormone and signaling molecule that mediates plant stress response (Per et al., 2018; Bizuneh, 2020; Muñoz-Hoyos and Stam, 2023; Xin et al., 2023). The KEGG analyses of DEGs in this study revealed a significant up-regulation of fatty acid metabolic pathways, which is consistent with previous reports (Ke et al., 2014; Yin et al., 2016), and cluster 2 of the network showed a strong association between the up-regulated metabolite ALA and several genes. All these results underscore the crucial roles of the metabolite ALA in apple resistance to AVC.

Betaine could enhance the stability of biomacromolecular structure and function, and reduce ROS accumulation by preserving the activity of ROS scavenging enzymes (Huang et al., 2020), which leads to improved plant resistance against various abiotic stresses such as metal ions, salt, low temperature, and drought (Li C. Y. et al., 2021; Jokinen et al., 2022). Moreover, exogenous betaine was added to enhance apple resistance against anthracnose leaf blight (Liu et al., 2022). In our study, we observed a significant accumulation of the betaine in the Glycine, serine, and threonine metabolism pathway. Combining our findings with previous research, we hypothesize that betaine may play a crucial role in apple resistance to *C. mali* invasion.

The phenylpropanoid pathway leads to synthesis of different types of flavonoid phytoalexins and phenolic compounds, which are involved in plant defense (Li P. Q. et al., 2021). The pathway starts with phenylalanine, which can be converted into aromatic compounds and phenylpropanoids in response to disease resistance (Geng et al., 2020). In addition, phenylalanine ammonia-lyase (PAL) is a crucial enzyme in secondary phenylalanine metabolism and is widely studied in plant responses to biotic stresses (Kurihara and Yamana, 2022; Mansoor et al., 2023). We observed that many DEGs and DAMs were enriched in the phenylalanine, tyrosine, and tryptophan biosynthesis pathways in both groups. Within these pathways, the expression of PAL gene MD11G1223500 was significantly increased after being infected. These metabolites and genes in the phenylpropanoid pathway were associated with the resistance of apple to *C. mali* infestation, and need further exploration.

The infection of apples by *C. mali* disrupts the cell structure and secretes pathogenic virulence factors. In response to *C. mali* infection, apples have evolved multiple strategies. Our study indicates that apples primarily resist *C. mali* infection by mitigating cell wall degradation, activating the PAL activity, and promoting the synthesis of defensive substances.

## 5 Conclusion

This study conducted a comparative analysis of the metabolome and transcriptome of ‘Jinhong’ apple branches between control and AVC inoculation. Pathway analysis of the apple resistance response revealed significant changes in several metabolic pathways, including galactose metabolism, fructose and mannose metabolism, phenylalanine, tyrosine and tryptophan biosynthesis, glycine, serine and threonine metabolism, and isoquinoline alkaloid biosynthesis, during the *C. mali* inoculation. Moreover, the transcriptional and metabolic patterns of the affected tissues were altered. The production and accumulation of several enzymes and metabolites, including β-Gal, UGP-glucose, ALA, betaine, and PAL may play a crucial role in the presentation of apples when challenged by *C. mali*. These results shed light on the potential molecular mechanisms underlying the infection of AVC in ‘Jinhong’ branches, further exploration of DEGs and DEMs are in need in future work.

## Data availability statement

The datasets presented in this study can be found in online repositories. The names of the repository/repositories and accession number(s) can be found in the article/Supplementary material.

## Author contributions

JZ: Formal analysis, Investigation, Writing – original draft, Methodology. YG: Data curation, Software, Writing – original draft. ZL: Resources, Writing – review & editing, Project administration. YS: Methodology, Writing – original draft. PS: Funding acquisition, Methodology, Project administration, Supervision, Validation, Writing – review & editing.

## Footnotes

^1^https://www.metaboanalyst.ca

^2^https://bio-cloud.aptbiotech.com

^3^https://cloud.metware.cn

^4^https://www.majorbio.com

^5^http://www.omic smart.com

^6^http://www.bioinformatics.com.cn
